# Supplementary material for: Stable antivortices in multiferroic ε-Fe2O3 with the coalescence of misaligned grains
Source: Nat Commun. 2025 Jan 7;16:440. doi: 10.1038/s41467-025-55841-x (PMC11704318; doi:10.1038/s41467-025-55841-x)
Supplement: Supplementary file 1 — Supplementary Information [file 41467_2025_55841_MOESM1_ESM.docx]

**Supporting Information for**

**Stable antivortices in** **multiferroic *ε*-Fe_2_O_3_ with the coalescence of misaligned grains**

Wuhong Xue^1,^^#,*^, Tao Wang^1,#^, Huali Yang^2,#^, Huanhuan Zhang^3^, Guohong Dai^4^, Sheng Zhang^1^, Ruilong Yang^1^, Zhiyong Quan^1^, Run-Wei Li^2^, Jin Tang^3*^, Cheng Song^5*^ & Xiaohong Xu^1*^

^1^Key Laboratory of Magnetic Molecules and Magnetic Information Materials of Ministry of Education & School of Chemistry and Materials Science of Shanxi Normal University, Taiyuan 030031, China

^2^CAS Key Laboratory of Magnetic Materials and Devices, Ningbo Institute of Materials Technology and Engineering, Chinese Academy of Sciences, Ningbo 315201, China

^3^School of Physics and Optoelectronic Engineering, Anhui University, Hefei 230601, China

^4^School of Physics and Materials Science & Institute of Space Science and Technology, Nanchang University, Nanchang 330031, China

^5^School of Materials Science and Engineering, Tsinghua University, Beijing 100084, China

^*^Corresponding authors: [xuewuhong@sxnu.edu.cn](mailto:xuewuhong@sxnu.edu.cn); [jintang@ahu.edu.cn](mailto:jintang@ahu.edu.cn); [songcheng@mail.tsinghua.edu.cn](mailto:songcheng@mail.tsinghua.edu.cn); [xuxh@sxnu.edu.cn](mailto:xuxh@sxnu.edu.cn)

^#^These authors contributed equally.

The PDF file includes:

Supplementary Note

Supplementary Fig.1 to 21

Supplementary Table 1

**Table of Contents**

**Note S1.** Procedures and parameter settings for the MFM experiments.

**Fig. S1.** Schematic diagram of the mechanism for creating vortex or antivortex.

**Fig. S2.** Schematic diagram of the mechanism for creating antivortex in 2D polycrystals.

**Fig. S3.** Schematic illustration for the growth of 2D *ε*-Fe_2_O_3_ by a CVD method.

**Fig. S4.** Formation of *ε*-Fe_2_O_3_ polycrystal on six-fold symmetric substrate.

**Fig. S5.** XPS and Raman characterizations of the as-grown *ε*-Fe_2_O_3_ nanosheets.

**Fig. S6.** Fe 2*p_3/2_* region of Fe 2*p* XPS spectrum.

**Fig. S7.** Lattice structures and aberration-corrected STEM images of *ε*-Fe_2_O_3_ nanosheet.

**Fig. S8.** AFM images of 2D *ε*-Fe_2_O_3_ polycrystal.

**Fig. S9.** Cross-sectional TEM images and EDS mapping image.

**Fig. S10.** Four-fold degeneracy of antivortices.

**Fig. S11.** Simulated total energy of four styles of antivortices.

**Fig. S12.** Antivortices in nanosheets with different lateral sizes and thicknesses.

**Fig. S13.** Simulated magnetizations and MFM images.

**Fig. S14.** Simulated stable phase diagram based on the thickness and lateral length.

**Fig. S15.** Simulated magnetic evolution by decreasing magnetocrystalline anisotropy.

**Fig. S16.** Total energy as a function of magnetocrystalline anisotropy.

**Fig. S17.** MFM images in a remnant state after applying external magnetic field.

**Fig. S18.** MFM images of *ε*-Fe_2_O_3_ nanosheet after cooling down to room temperature from various temperatures.

**Fig. S19.** *In-situ* temperature-variable MFM measurement.

**Fig. S20.** Environmental stability of antivortices confirmed by MFM results.

**Fig. S21.** Final stable phase triggered by in-plane pulsed field.

**Table S1.** The detailed information on Fe 2*p_3/2_* component peaks of Fe^3+^.

**Supplementary Note S1**

**The principles of MFM experiments.** Both AFM images and MFM phase images are acquired through MFM experiment. During the MFM experiment, tapping mode is first used to scan the sample surface to obtain topographical information (AFM image). Subsequently, lift mode is selected, where the tip is raised above the sample surface. The surface topography from the initial scan is incorporated into the lift scan process. During the lift scan, the tip and the sample are maintained at a constant distance, and magnetic interactions are detected during the second scan (MFM phase image).

**Procedures and parameter settings.**

1. Before installing the probe holder on the Dimension Icon scanner, the magnetically coated probe (MESP-V2, Bruker) is first magnetized using a permanent magnet. The magnetization of the tip is oriented along the axis of the tip (perpendicular to the sample surface). Subsequently, the MFM senses the force gradient from the vertical component of the sample's magnetic field.

2. After installing the magnetic probe onto the probe holder, set the cantilever drive frequency to 75 kHz, the lift height to 120 nm, and the scan rate to 1 Hz.

3. First trace/retrace: the cantilever tracks the surface topographies of the *ε*-Fe_2_O_3_ nanosheets; then the cantilever is raised to the lift scanning height; second trace/retrace: magnetic field information is measured by cantilever along the surface topography.


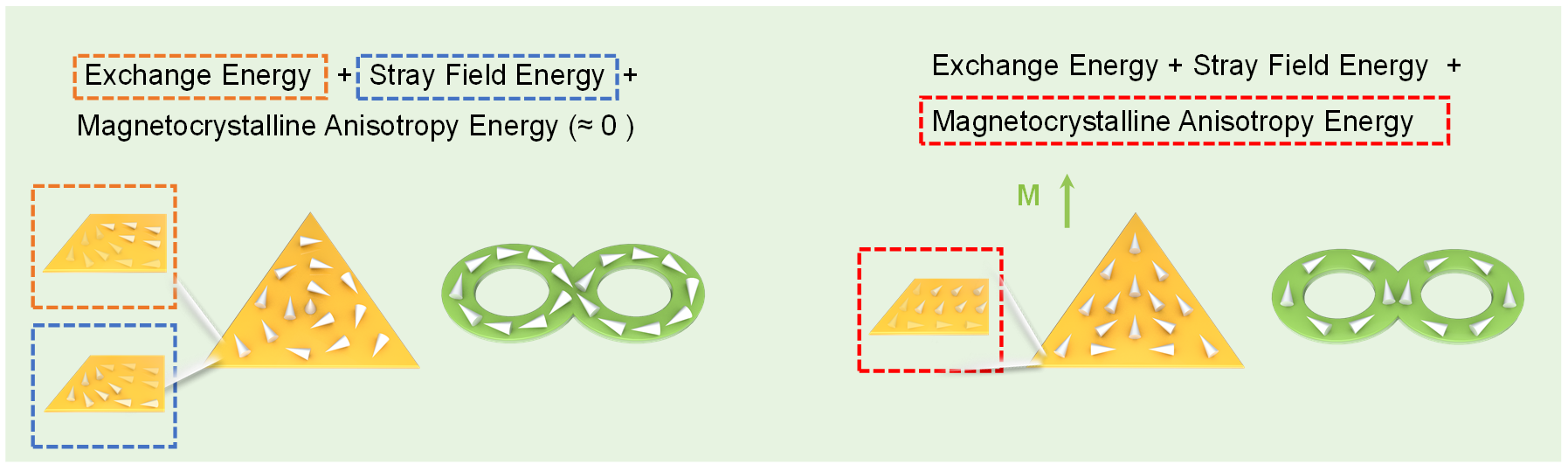


**Supplementary Fig. 1 |** **Schematic diagram of the mechanism for creating magnetic vortex or antivortex based on the principle of energy minimization.** Left panel displays the cases where magnetocrystalline anisotropy is ignored, using triangular and ‘∞-shaped’ structures as examples. White arrows represent the direction of magnetization circulation. The boxes on the left are locally enlarged images of the triangular structure to highlight the influence of exchange energy and stray field energy. The right panel displays the cases where the magnetocrystalline anisotropy cannot be ignored. “**M**” represents magnetization vector.

Generally, vortex and antivortex forms due to the principle of energy minimization. When the MCA of the magnets could be ignored, the exchange energy mainly competes with the stray field energy. For example, in a triangular structure, the magnetization circulating clockwise or counterclockwise around the center will minimize the magnetic charge at the boundary (highlighted by the blue dashed box), and the magnetic moment inside the structure tends to align parallelly to minimize exchange energy (highlighted by the yellow dashed box). At the center, the magnetization curls out of plane, minimizing the exchange energy with an increase in the stray field energy. Similarly, in the ‘∞-shaped’ structure, magnetic moments align along the edge of two adjacent rings and naturally forms two vortices to keep the overall energetically favored state. Two vortices with the same chirality generate opposite magnetic charges on the same edge of adjacent rings, reducing the stray field energy at the cost of increasing domain wall energy. After applying a specific sequence of external magnetic fields, a typical isolated antivortex could be generated. When MCA cannot be ignored, magnetization is aligned towards the easy magnetization axis to reduce magnetostatic energy. Magnetic single domain usually appears in relatively confined structures and transforms into multidomain as the size increases.


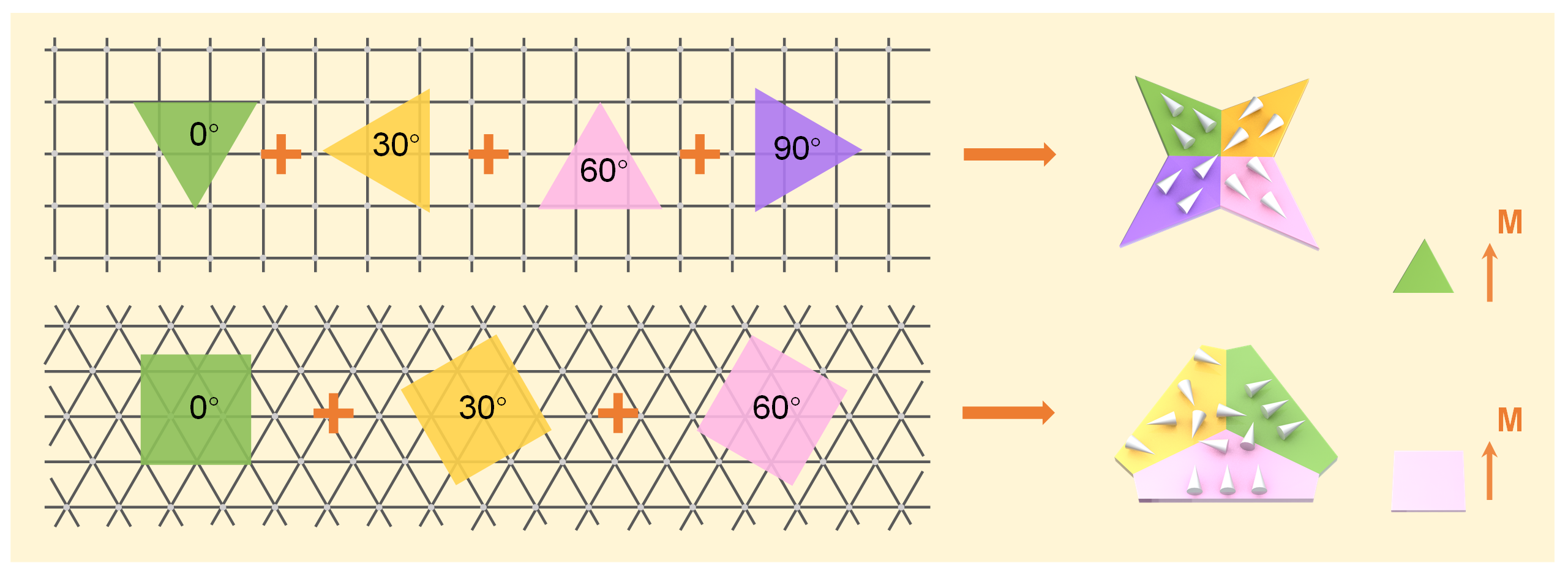


**Supplementary Fig. 2 | Schematic diagram for creating magnetic antivortex in 2D magnetic polycrystals.** Schematic of the equivalent low-energy alignments of threefold (fourfold) symmetric 2D materials on fourfold (six-fold) symmetric substrates, and the principle on the construction of the coalescence of these different oriented triangular or square grains to form geometrically highly symmetric polycrystals. “**M**” represents magnetization vector.

Multiple nucleations are ubiquitous in the chemical vapor deposition processes for 2D materials growth. For example, threefold symmetric 2D materials have four equivalent low-energy orientations on a fourfold symmetric substrate and fourfold symmetric 2D materials have three equivalent low-energy orientations on a six-fold symmetric substrate (left panel of **Supplementary Fig. 2**). Multiple 2D grains with different orientations can coalesce to form polycrystals with diverse shapes. The coalescence of four different oriented triangular grains on a fourfold symmetric substrate can form a cross-shaped structure, while the coalescence of three different oriented square grains on a six-fold symmetric substrate can form a truncated triangular structure (right panel of **Supplementary** **Fig. 2**). By selecting appropriate 2D materials and substrates, 2D polycrystals with highly symmetric in geometry can be fabricated. When these 2D materials have appropriate magnetocrystalline anisotropy constants, they can serve as ideal platforms for constructing magnetic antivortex texture.


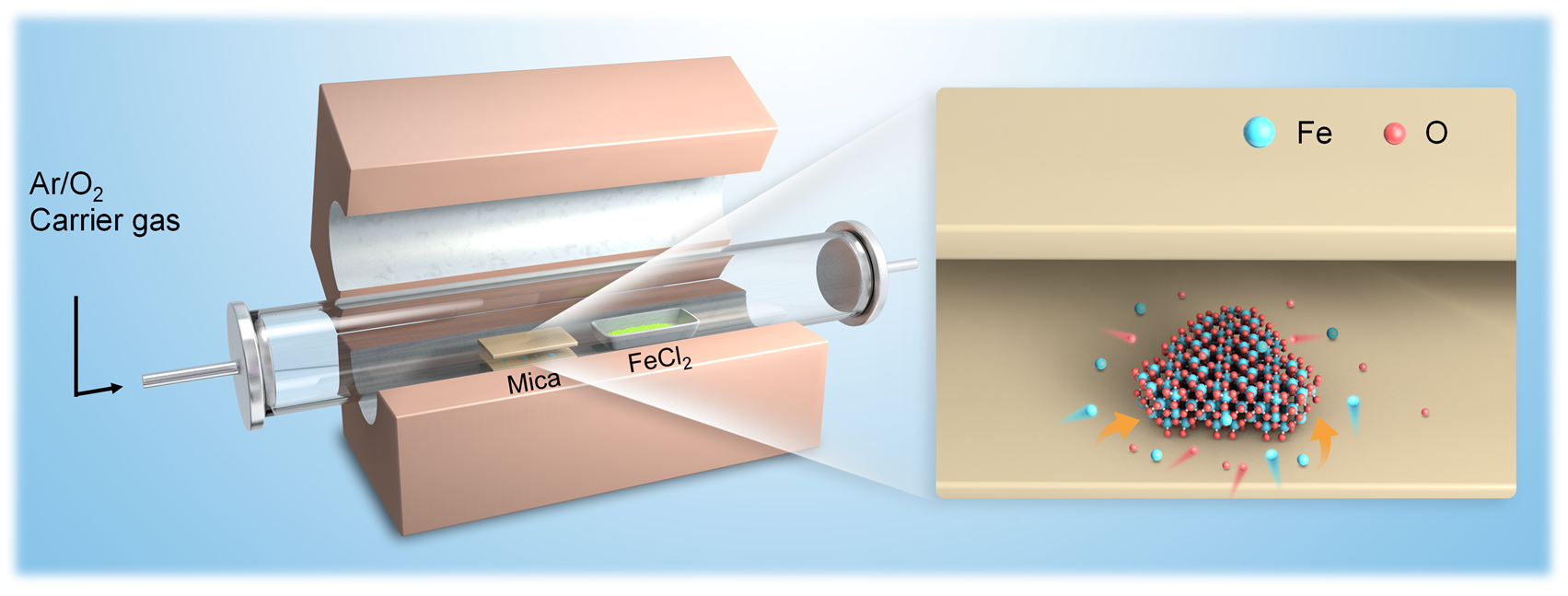


**Supplementary Fig. 3 | Schematic illustration of the confined CVD setup for the growth of truncated triangular 2D *ε*-Fe_2_O_3_.** The orange arrows represent the landing and docking of Fe and O atoms on mica substrate.


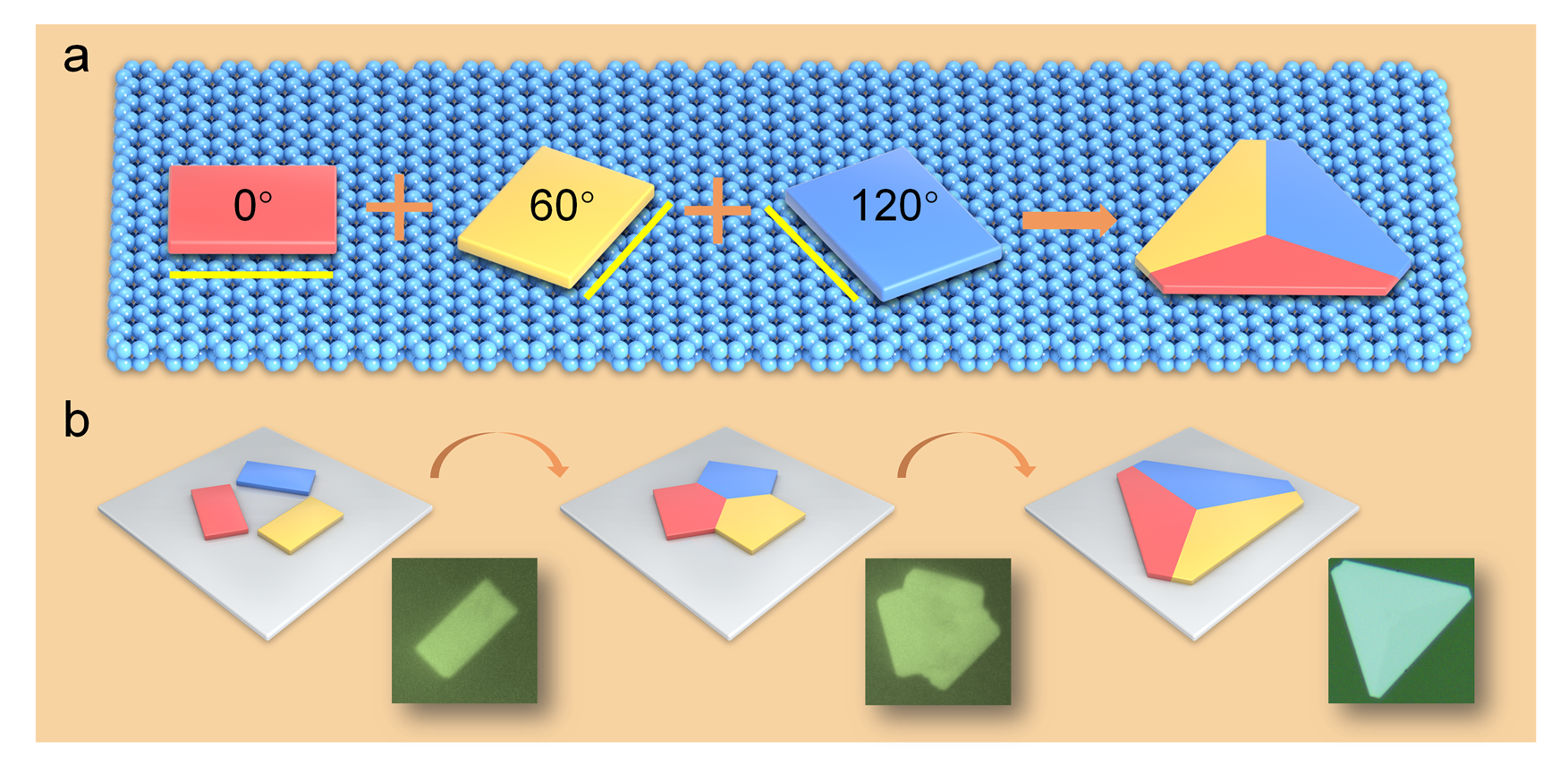


**Supplementary Fig. 4 |** **Schematic diagram of growth process of truncated triangular *ε*-Fe_2_O_3_ polycrystal. a** Schematic diagram of a truncated triangular polycrystal formed by the coalescence of rectangular grains with three different orientations. The relative orientation angle of each grain is provided. The edges of 2D *ε*-Fe_2_O_3_ that are preferentially aligned to the high symmetric directions of the substrates are indexed by yellow lines. **b** Sketch of the growth process of a 2D *ε*-Fe_2_O_3_ polycrystal. Inset: Corresponding optical micrograph images of *ε*-Fe_2_O_3_ at the different growth stages.


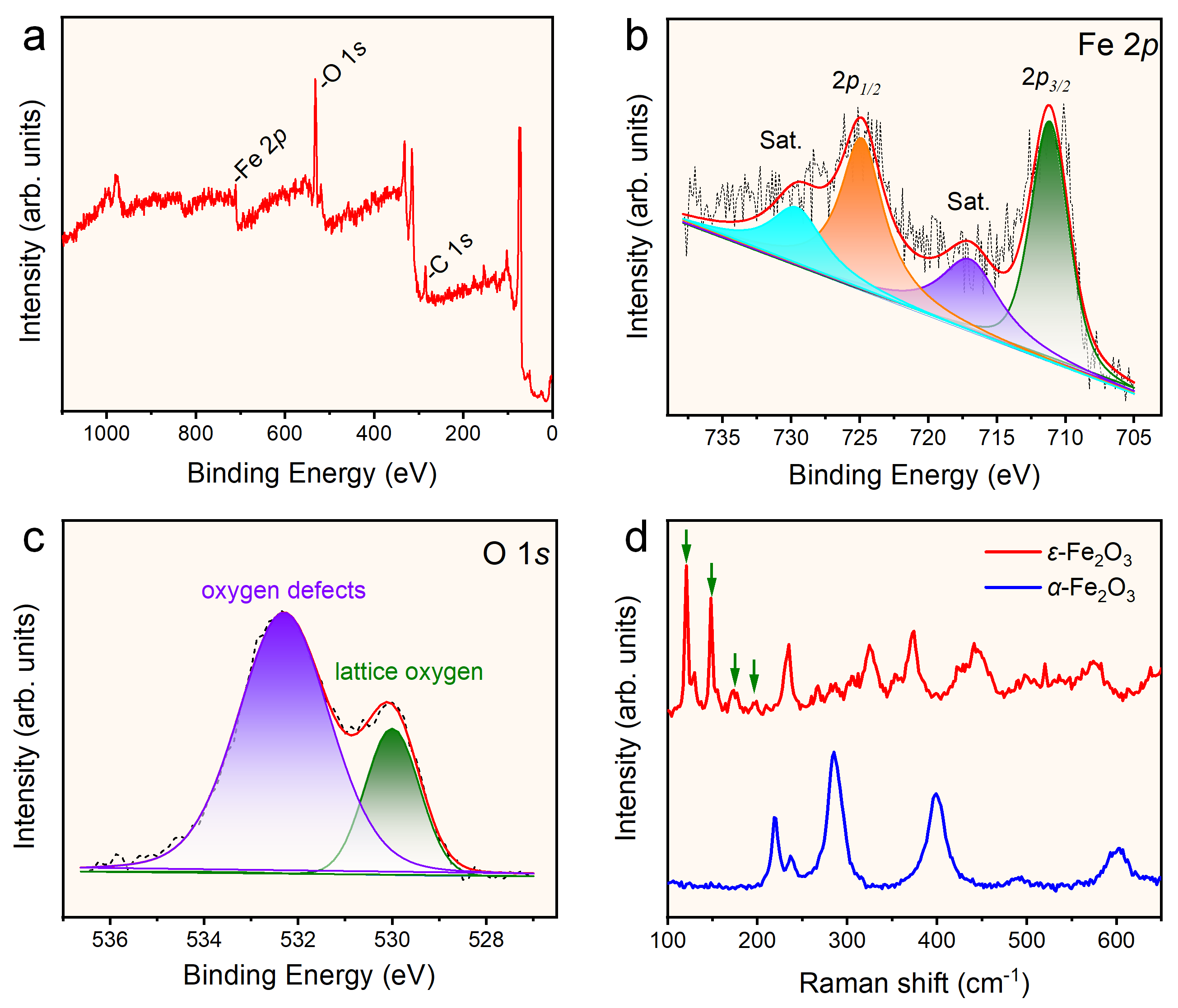


**Supplementary Fig. 5 | XPS and Raman characterizations of the as-grown *ε*-Fe_2_O_3_ nanosheets.** **a** XPS survey spectrum of the transferred *ε*-Fe_2_O_3_ nanosheets onto Pt/SiO_2_/Si. **b, c** High resolution spectrum of Fe 2*p* (**b**) and O 1*s* (**c**). The binding energies at ~724.6 and ~711.1 eV are attributed to Fe^3+^, and no Fe^2+^ is found in these nanosheets from the XPS results. **d** Raman spectra of *ε*- and *α*-Fe_2_O_3_ crystals. *ε*-Fe_2_O_3_ exhibits four distinct characteristic peaks between 100 and 200 cm^−1^ marked by green arrows, while *α*-Fe_2_O_3_ has no active Raman modes within this range.


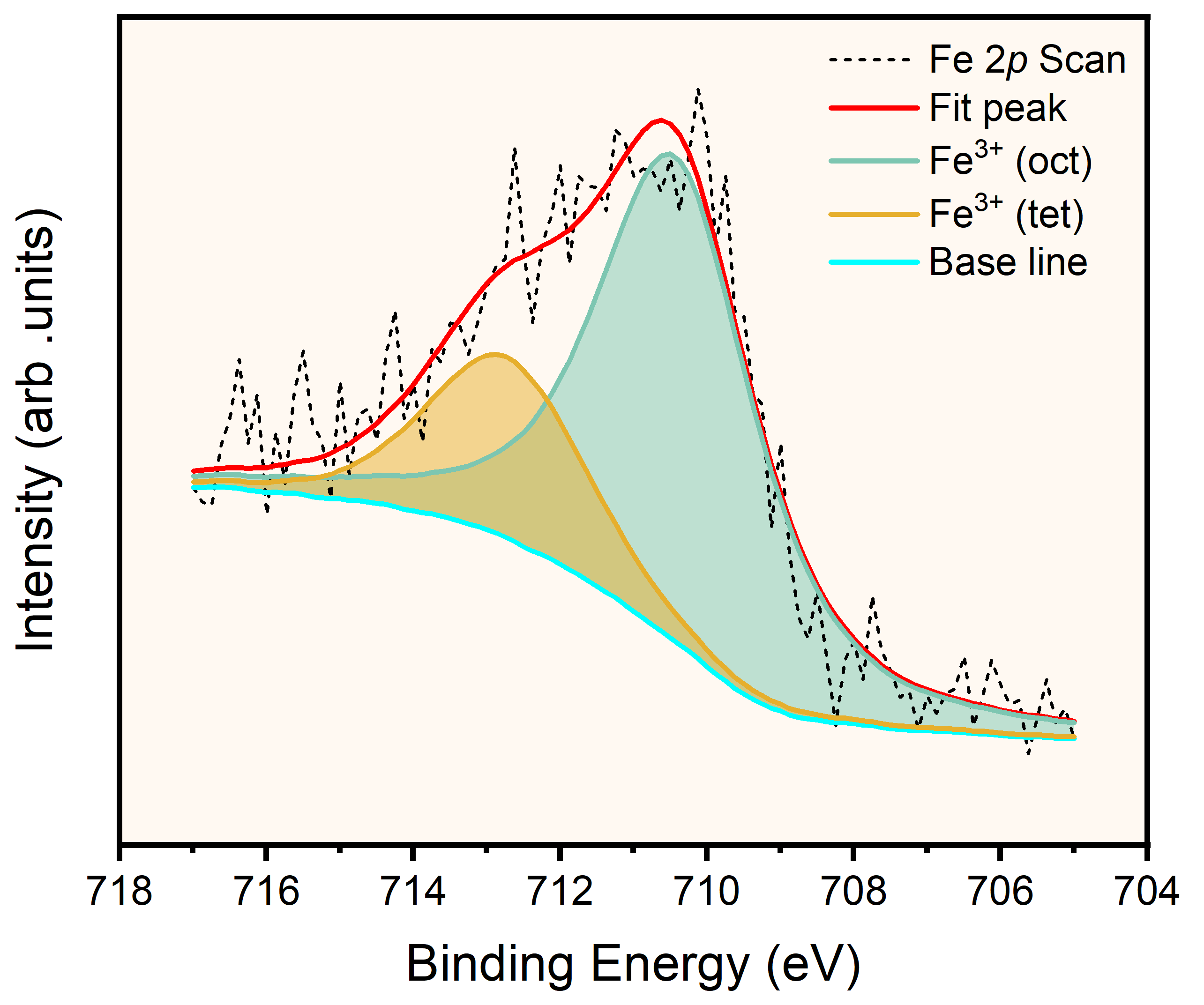


**Supplementary Fig. 6 | Fe 2*p_3/2_* region of Fe 2*p* XPS spectrum.** Contribution from the Fe^3+^(oct) and Fe^3+^(tet) cations to the Fe 2*p_3/2_* peak are shown by color-filled deconvoluted peaks.

It is known that Fe^3+^ in *ε*-Fe_2_O_3_ is distributed on both octahedral (oct) and tetrahedral (tet) sites. In the Fe 2*p_3/2_* region, the peak located at 711.1 eV is deconvoluted into two peaks. The binding energies of the Fe^3+^(oct) and Fe^3+^(tet) peaks are 710.4 eV and 712.5 eV, respectively, which are consistent with the values reported in the literature^1,2^. The fitted yields a Fe^3+^(oct)/Fe^3+^(tet) ratio of 2.98 which is in reasonable agreement with the expected value of 3 for the *ε*-Fe_2_O_3_ crystal structure. The related parameters of each component peak, including their positions, relative areas, full width at half maximum (FWHM), and squared deviation (*χ*²), are listed in **Supplementary Table 1**.

**Supplementary Table 1 |** Detailed information on Fe 2*p_3/2_* component peaks of Fe^3+^ in octahedral (oct) and tetrahedral (tet) sites obtained from the Fe 2*p_3/2_* region in XPS spectrum of *ε*-Fe_2_O_3_.

| Deconvoluted  Region | Peak Position (eV) | Attribution | Relative Area | FWHM (eV) | *χ*^2^ | Peak Ratio |
| --- | --- | --- | --- | --- | --- | --- |
| Fe 2*p_3/2_* | 710.4 | Fe^3+^ (oct) | 2899.7 | 2.6 | 1.27 | 2.98:1 |
|  | 712.5 | Fe^3+^ (tet) | 973.1 | 2.6 |  |  |


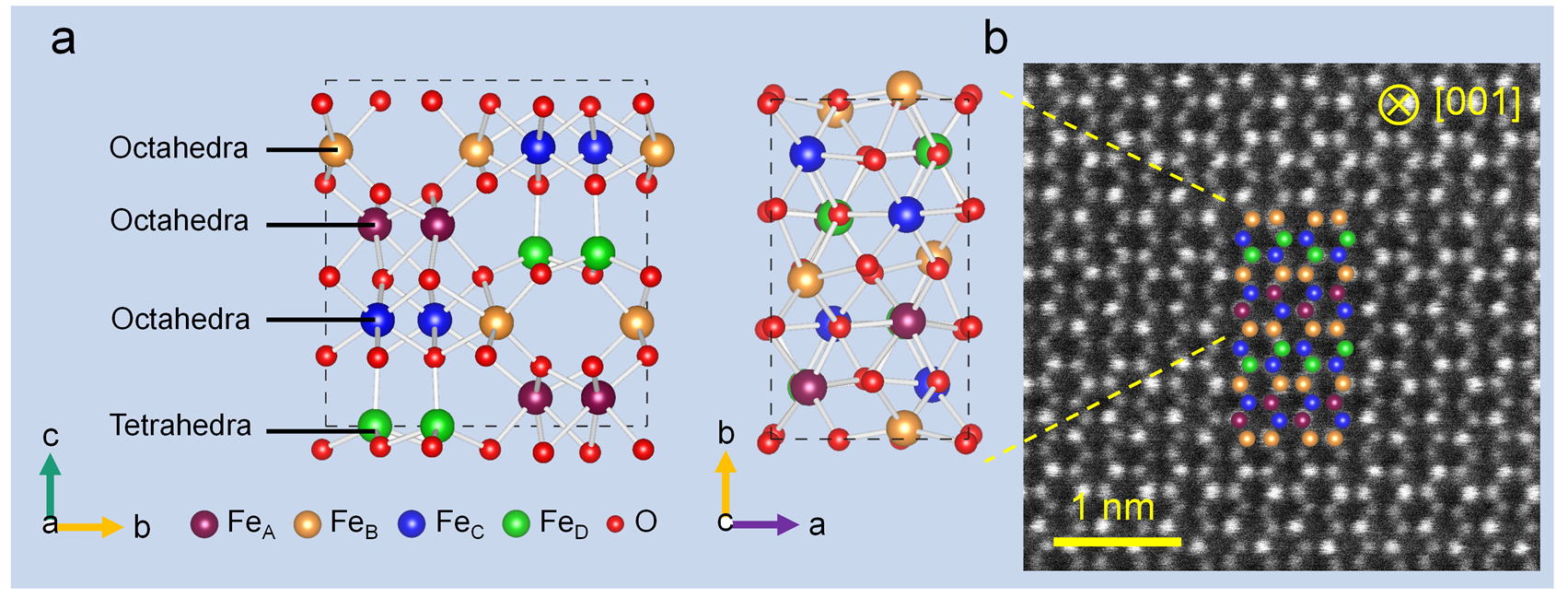


**Supplementary Fig. 7 | Crystal structure of *ε*-Fe_2_O_3_. a** Lattice structures of *ε*-Fe_2_O_3_ along [100] (side view) and [001] crystal axes (top view). In the structural model, correspondingly colored spheres identify different Fe^3+^ sites: octahedral sites (Fe_A_, Fe_B_, and Fe_C_) and tetrahedral site (Fe_D_). **b** Aberration-corrected STEM images of *ε*-Fe_2_O_3_ nanosheet.

As shown in **Supplementary** **Fig. 7b**, a perfect lattice structure is observed with almost no visible defects. The atomic arrangement perfectly matches the lattice structure of *ε*-Fe_2_O_3_ along the [001] crystal axis. The Fe^3+^ ions located at the Fe_A_, Fe_B_, Fe_C_, and Fe_D_ sites are represented by spheres with different colors, and it is evident that their ratio among each other is 1:1:1:1. The ratio of octahedral sites to tetrahedral is 3:1, which is consistent with the XPS results.


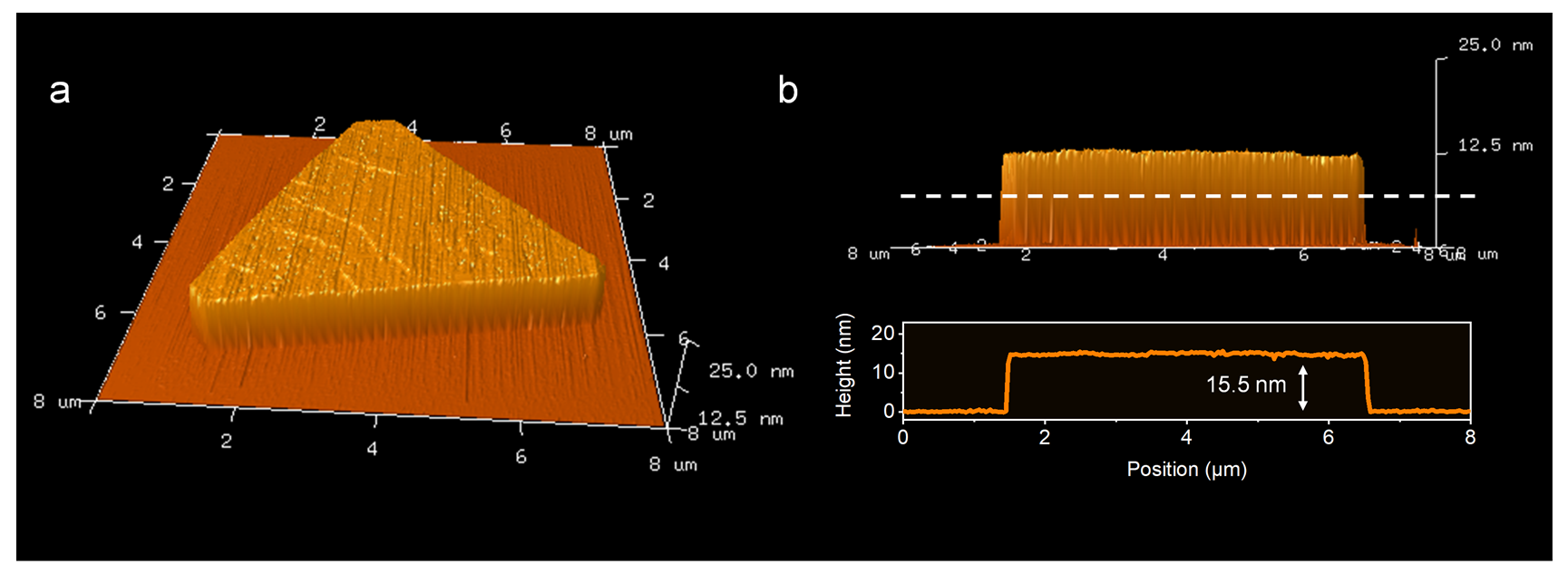


**Supplementary Fig. 8 | AFM image of 2D *ε*-Fe_2_O_3_ polycrystal. a** 3D morphology of the *ε*-Fe_2_O_3_ nanosheet in Figure 2. **b** Side view of morphology in (**a**) (upper panel), with the height profiles along the white dashed lines (lower panel).


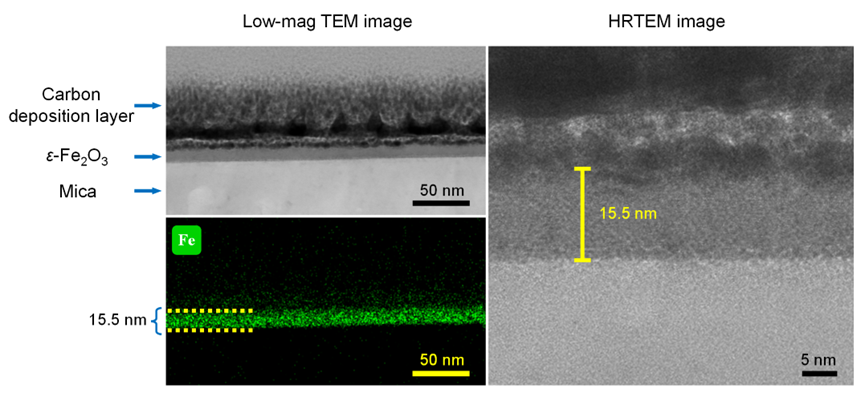


**Supplementary Fig. 9 | TEM images of *ε*-Fe_2_O_3_ sample in Figure 2.** Cross-sectional TEM images with low- and high-magnification of the *ε*-Fe_2_O_3_ nanosheet, and corresponding EDS mapping image of Fe, showing the homogeneous chemical constitution distributions.


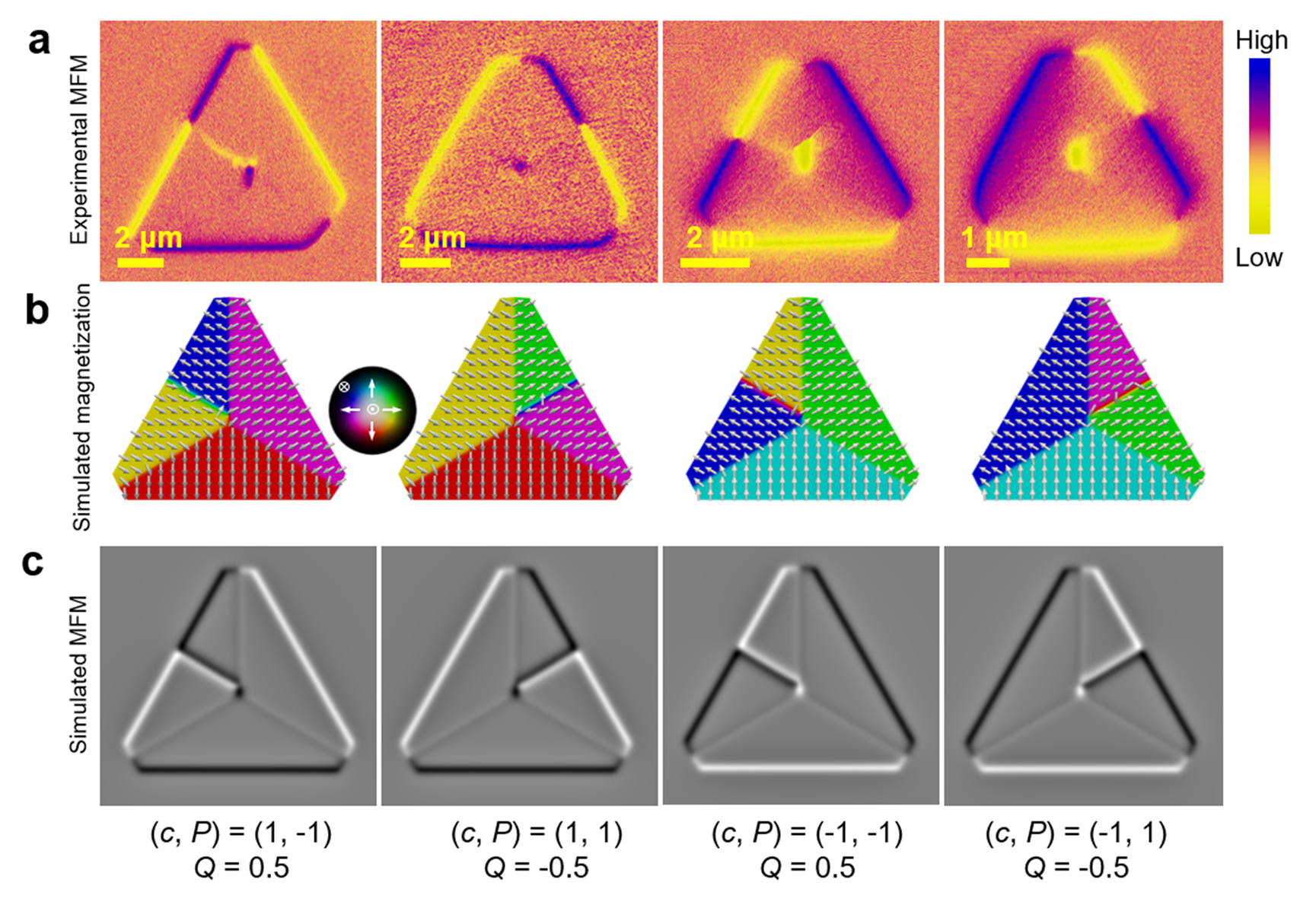


**Supplementary Fig. 10 | Four-fold degeneracy of antivortices.** MFM phase images (**a**), simulated magnetization distribution (**b**), and simulated MFM phase images (**c**) of four energetically degenerated magnetic antivortex states. The direction of the magnetic moment is shown by different colors in color-coded legend.


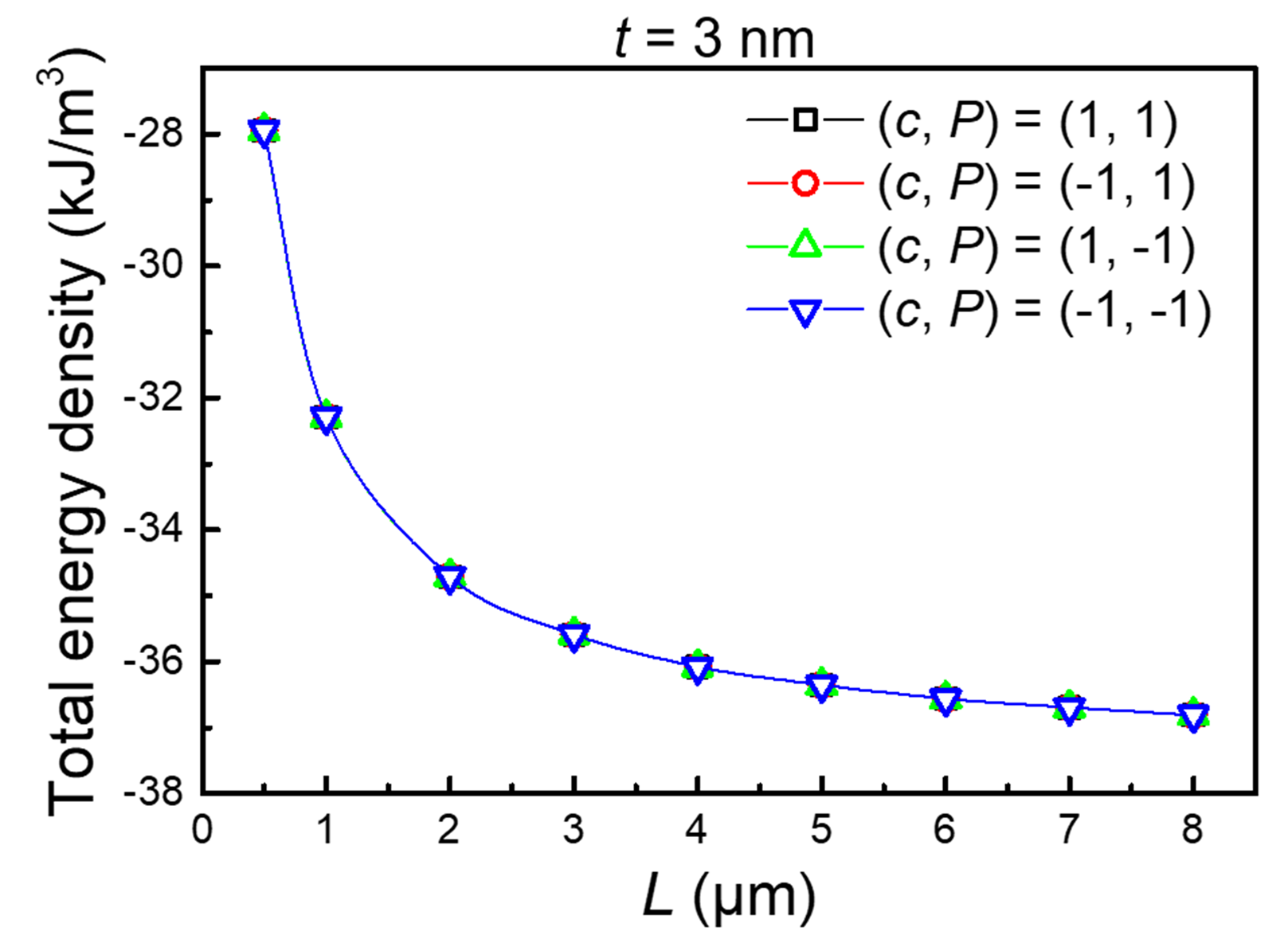


**Supplementary Fig. 11 |** **Four-fold degeneracy of antivortices in the truncated triangular multiferroic *ε*-Fe_2_O_3_ polycrystals.** Simulated total energy of four styles of antivortices denoted by colored hollow symbols.

**
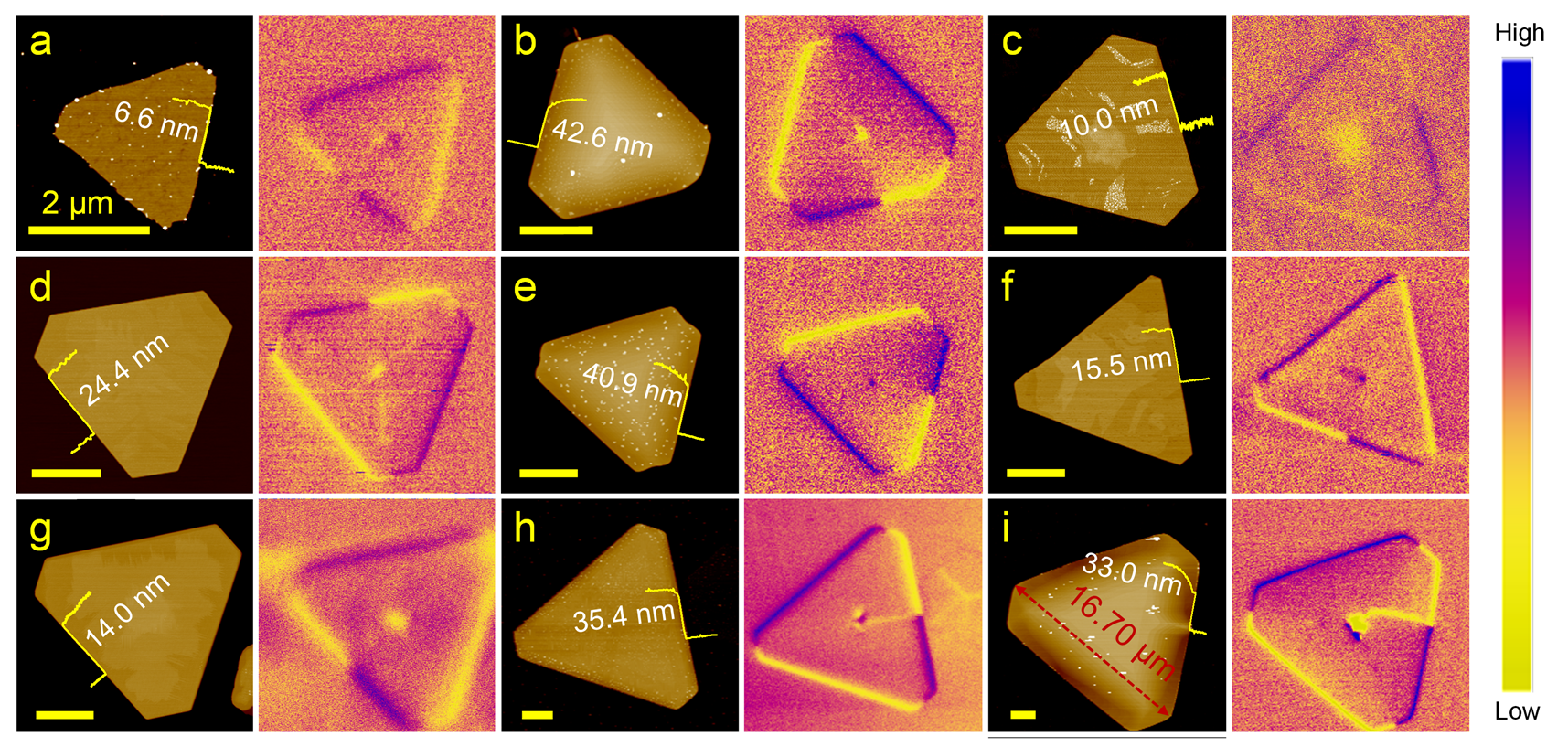
**

**Supplementary Fig. 12 | Magnetic antivortex stabilized in *ε*-Fe_2_O_3_ nanosheets with different thickness and lateral size. a-i** AFM images and corresponding MFM phase images of 9 *ε*-Fe_2_O_3_ nanosheets with different thicknesses arranged in ascending order of lateral size. The scale bar is 2 μm.


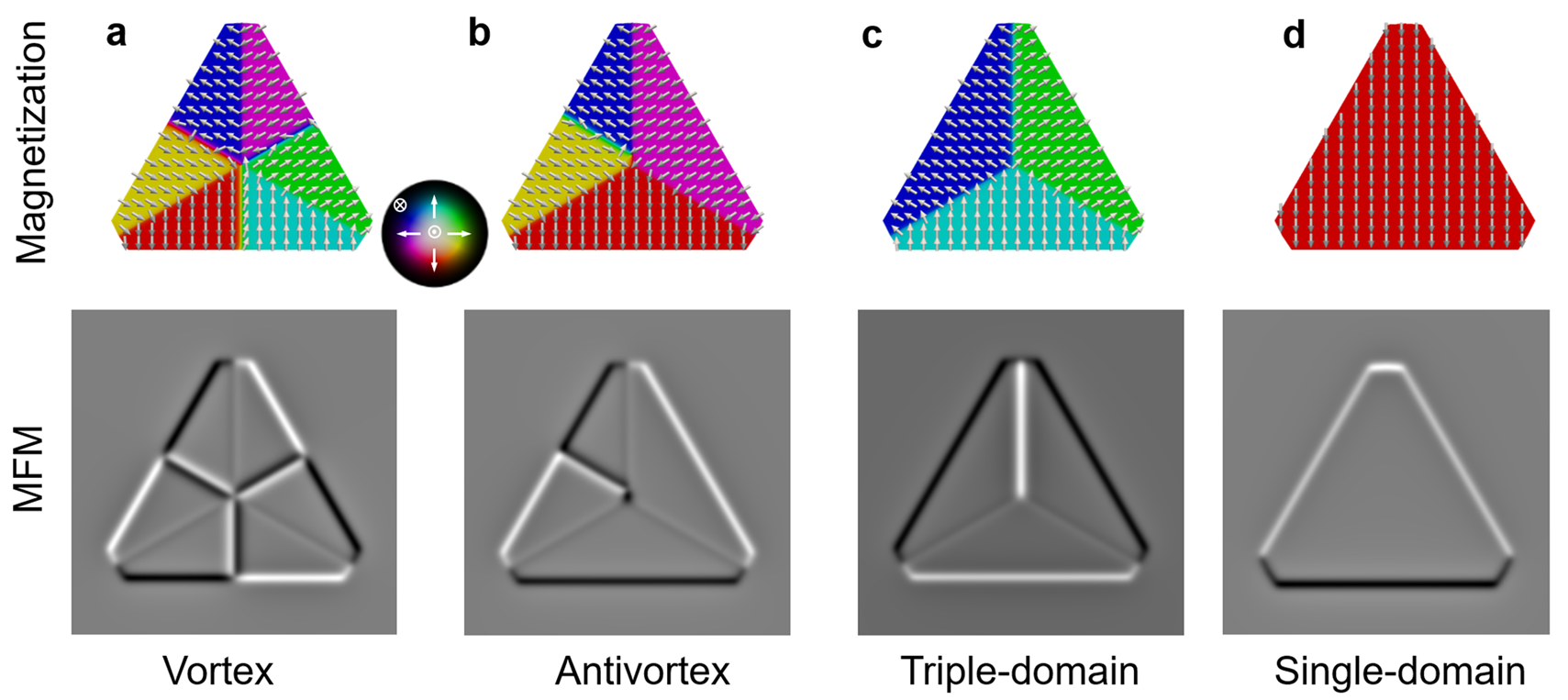


**Supplementary Fig. 13 |** **Simulated magnetic structures of 2D *ε*-Fe_2_O_3_ polycrystals.** Simulated magnetizations and MFM images of the vortex (**a**), antivortex (**b**), triple-domain (**c**), and single-domain (ferromagnet) (**d**). The direction of the magnetic moment is shown by different colors in color-coded legend.


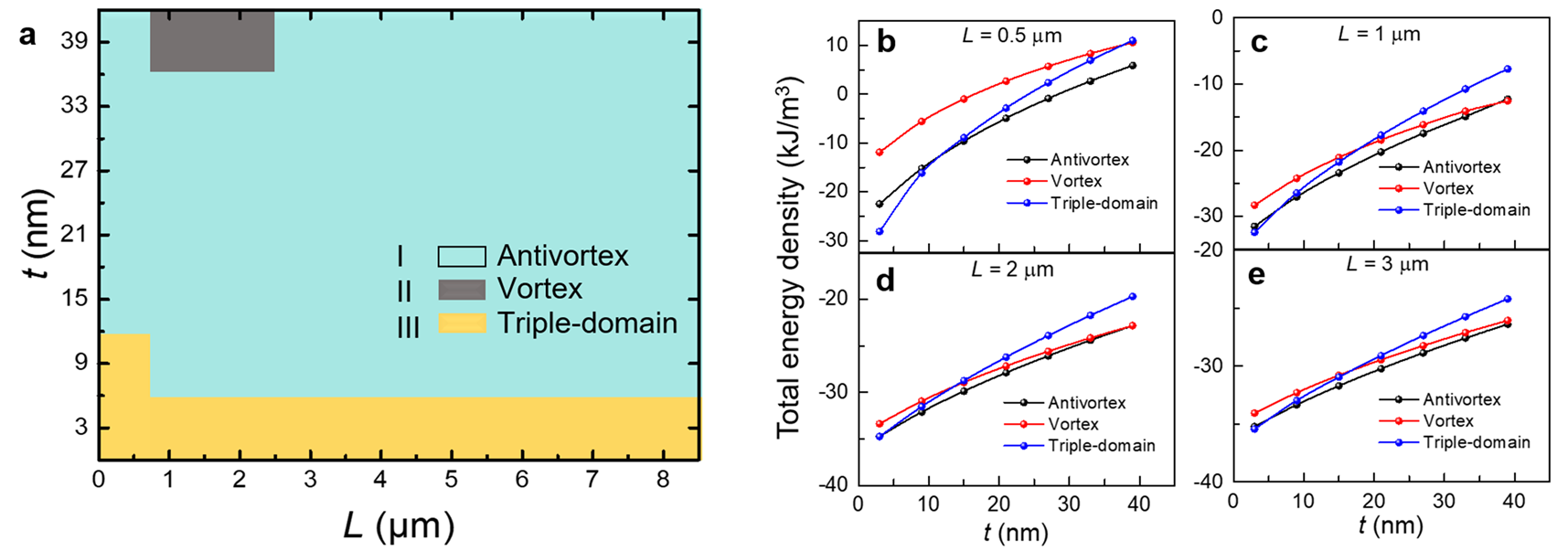


**Supplementary Fig. 14 |** **Simulated stable phase diagram based on the thickness and lateral length at zero fields.** **a** Simulated stable phase diagram as a function of geometrical size. Hollow black rectangle corresponds to the antivortex state. Filled dark grey and yellow rectangles correspond to the vortex, and triple-domain state, respectively. **b-e** Dependence of total energy density on thickness *t* for a fixed length *L* at 0.5, 1, 2, and 3 μm.


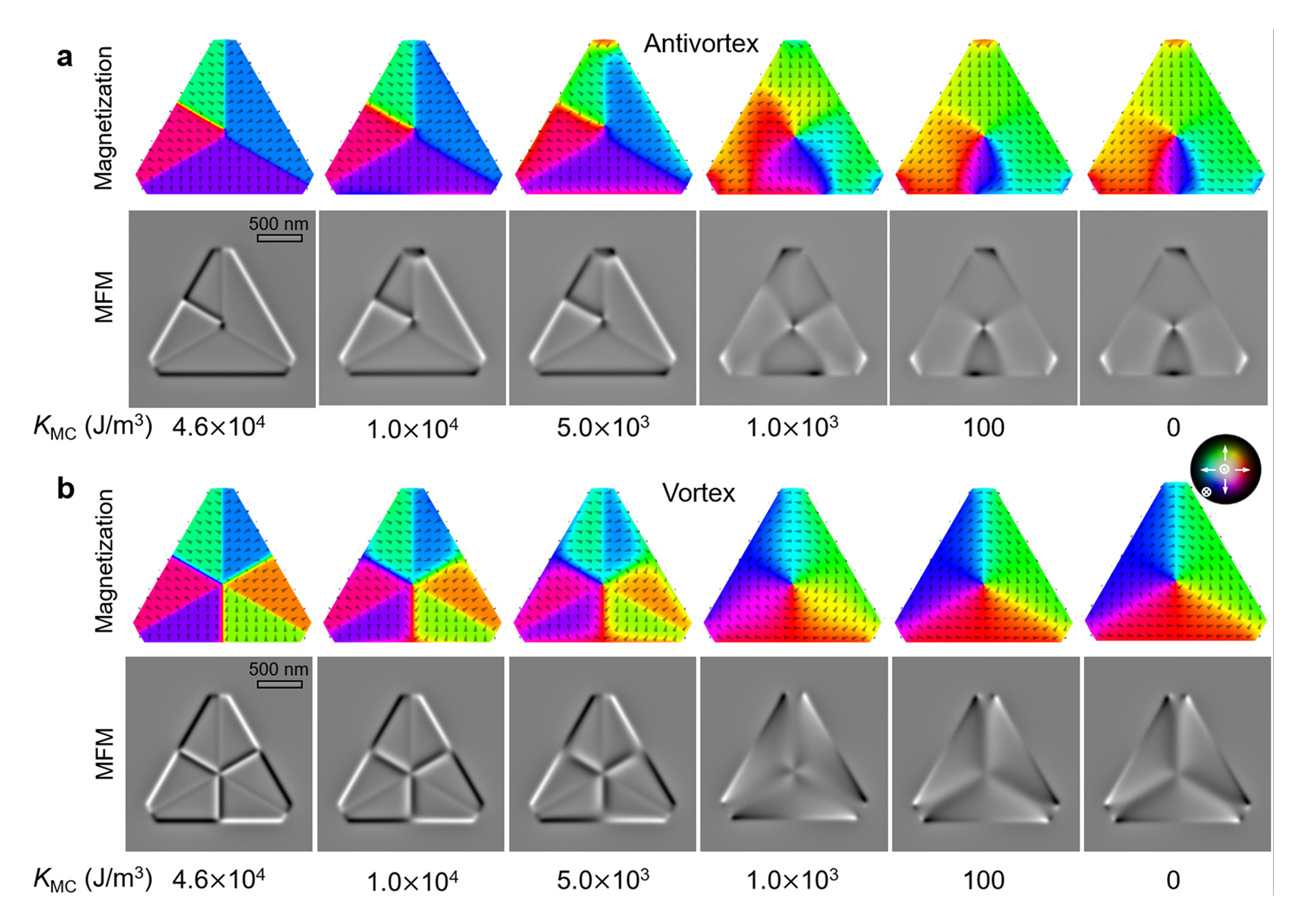


**Supplementary Fig. 15 | Simulated magnetic evolution by decreasing the magnetocrystalline anisotropy.** **a** Magnetic evolution from an initial antivortex. **b** Magnetic evolution from an initial vortex. *t* = 3 nm. *L* = 2 μm. The direction of the magnetic moment is shown by different colors in color-coded legend.


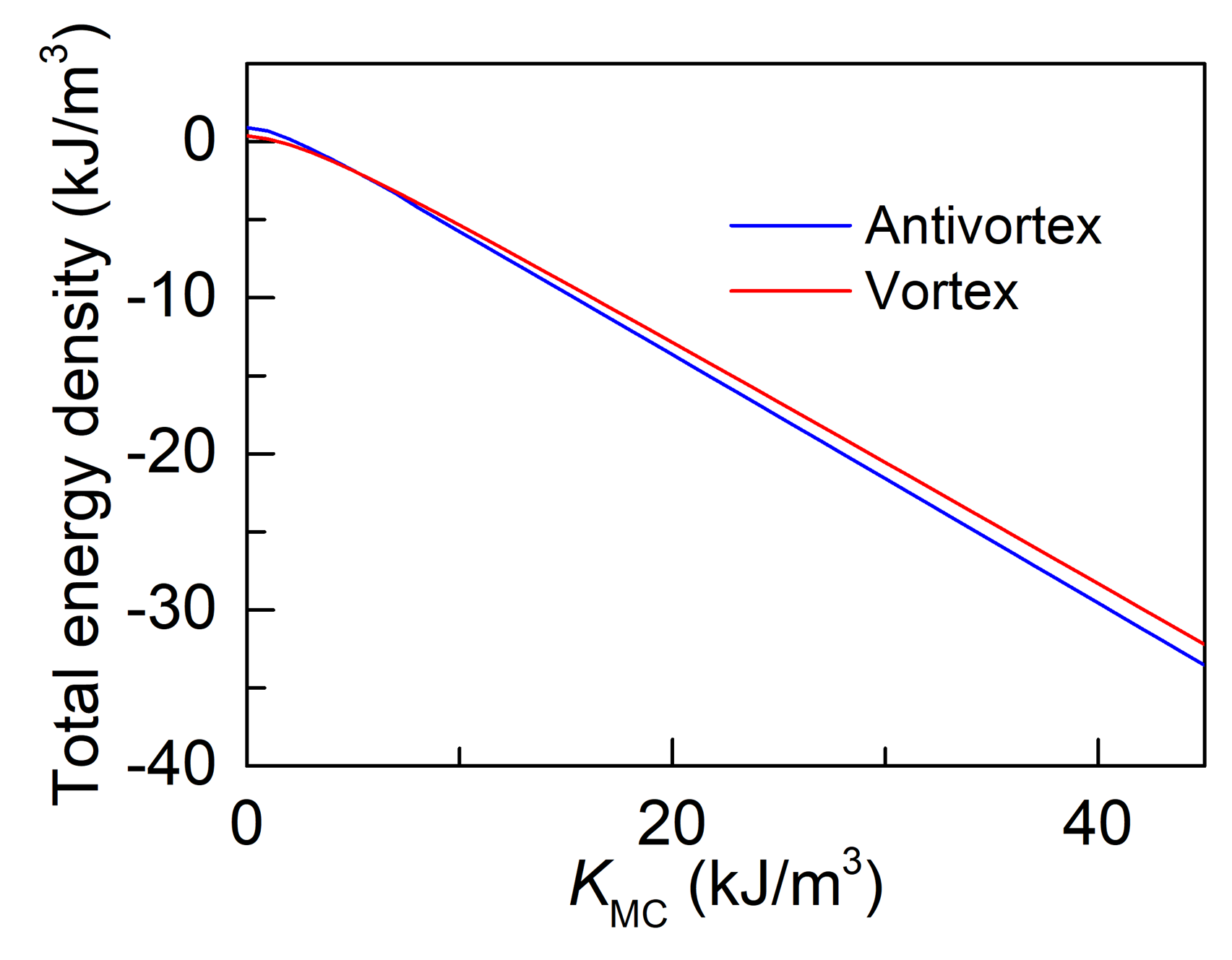


**Supplementary Fig. 16 | Total energy density versus magnetocrystalline anisotropy.** Total energy as a function of magnetocrystalline anisotropy for antivortex and vortex.

**
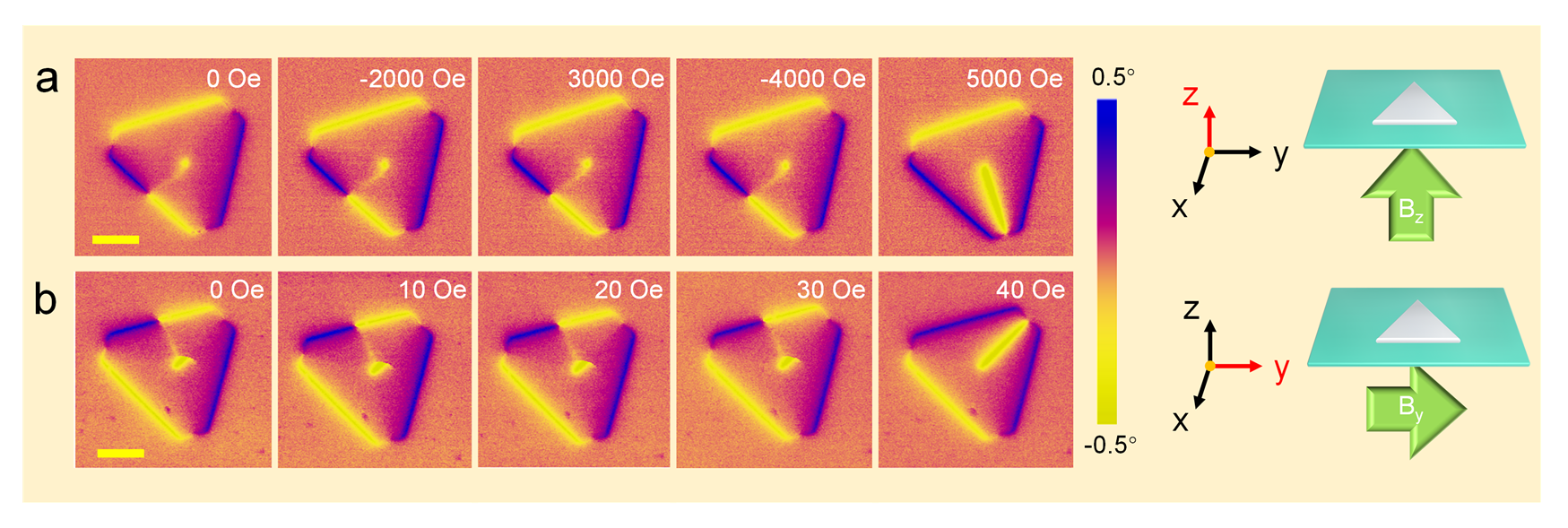
**

**Supplementary Fig. 17 | Evolution of magnetic antivortices after applied magnetic field. a, b** Series of MFM images in a remnant state after applying an external magnetic field along the out-of-plane z direction (**a**) and in-plane y direction (**b**). The scale bar is 2 μm. Configurations of the applied magnetic field **B** are displayed on the right panel.

**
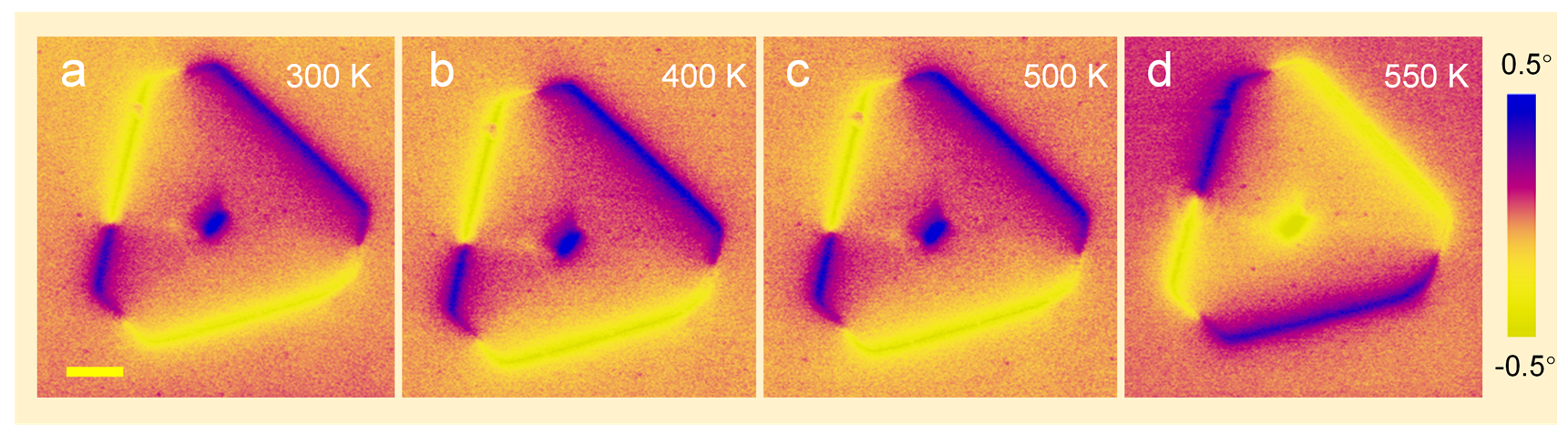
**

**Supplementary Fig. 18 | Reconstruction of magnetic antivortex after annealing above the magnetic transition temperature. a-d** MFM phase images of a magnetic antivortex measured at room temperature after heating at 300 K (**a**), 400 K (**b**), 500 K (**c**), and 550 K (**d**) for 10 min, respectively. The scale bar is 1 μm.


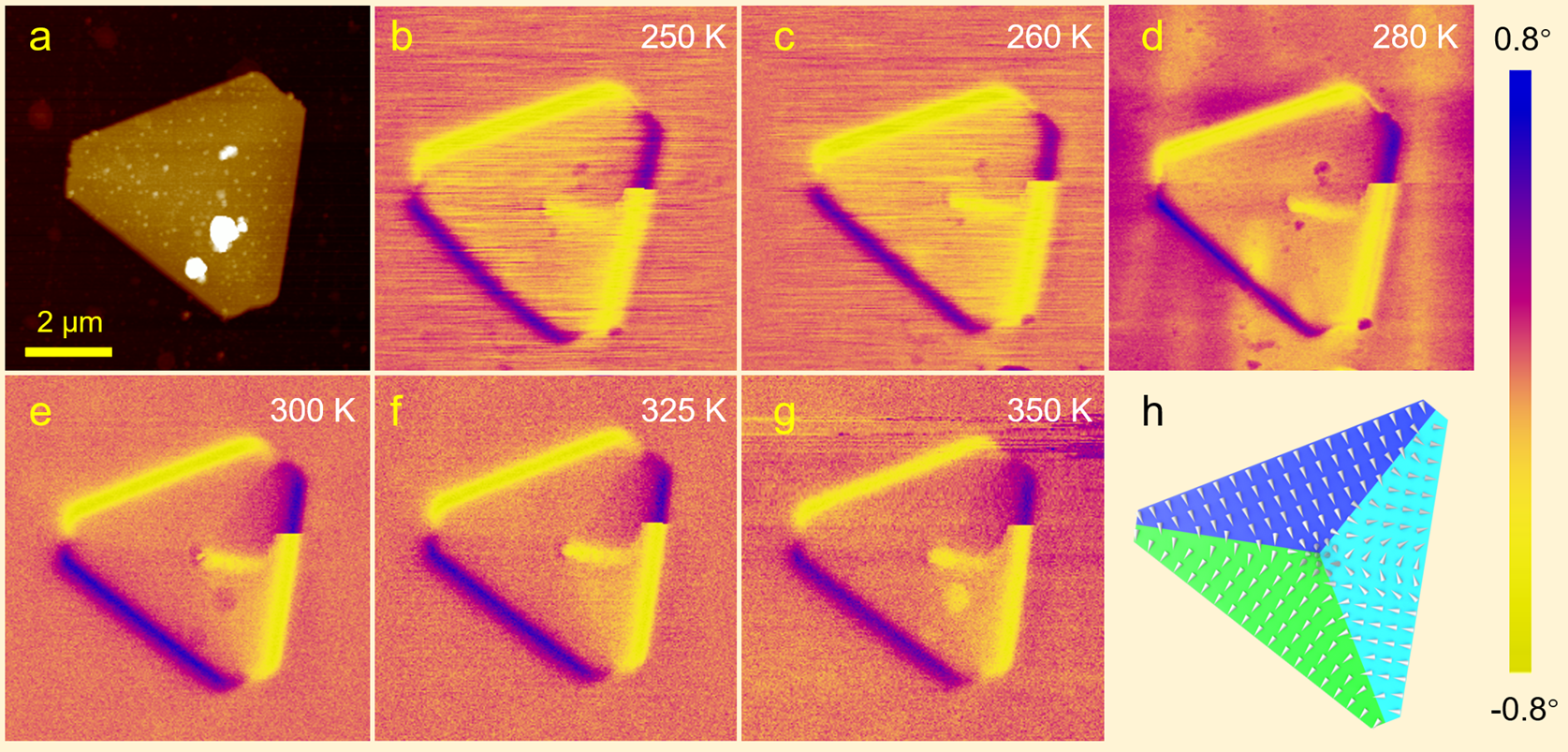


**Supplementary Fig. 19 | *In-situ* temperature-variable MFM measurement. a** AFM image of *ε*-Fe_2_O_3_ nanosheet. **b-g** MFM phase images recorded at 250, 260, 280, 300, 325, and 350 K, respectively. **h** The corresponding model of magnetization circulation.


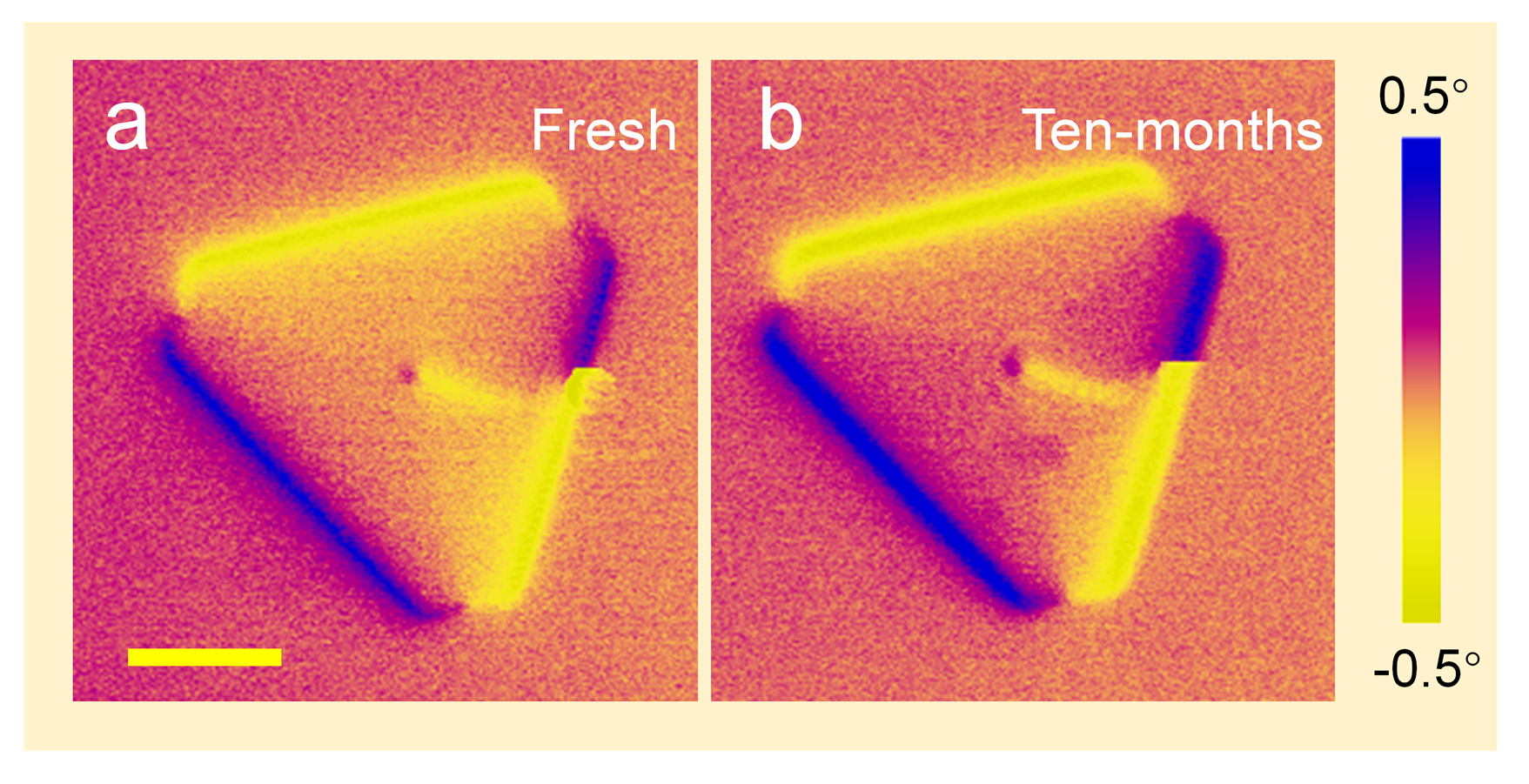


**Supplementary Fig. 20 | Environmental stability of magnetic antivortex in 2D *ε*-Fe_2_O_3_ polycrystals.** MFM phase images of a freshly synthesized (**a**) and ten-months aged (**b**) *ε*-Fe_2_O_3_ polycrystalline nanosheet. Both of them were measured at room temperature. The scale bar is 2 μm.


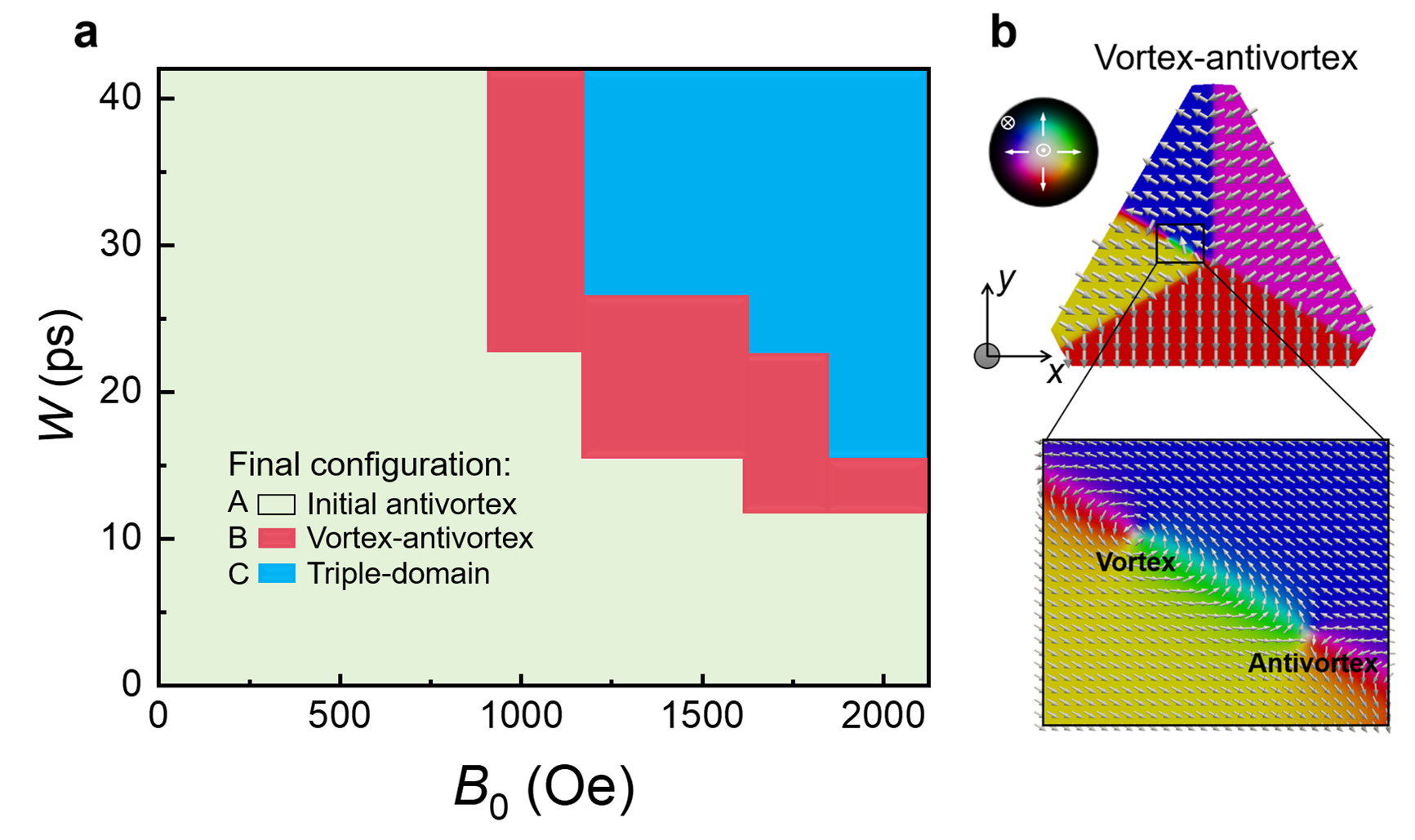


**Supplementary Fig. 21 | Final stable phase triggered by in-plane pulsed field.** **a** A stable phase diagram varying with the peak field **B**_0_ and *w*. Hollow black rectangle corresponds to the initial antivortex state. Filled dark red and blue rectangles correspond to the vortex-antivortex, and triple-domain state, respectively. **b** Magnetic configuration of the vortex-antivortex pair. The direction of the magnetic moment is shown by different colors in color-coded legend.

**Supplementary References**

1. Chandra, S. et al. Epitaxial magnetite nanorods with enhanced room temperature magnetic anisotropy. *Nanoscale* **9**, 7858-7867 (2017).
2. Jang, M. S. et al. Formation of Y_3_Fe_5_O_12_ matrix including *ε*-phase Fe_2_O_3_ with the giant coercive field via optimized sol–gel method. *J. Magn. Magn. Mater.* **552**, 169218 (2022).
