# Supplementary material for: Stable antivortices in multiferroic ε-Fe2O3 with the coalescence of misaligned grains
Source: Nat Commun. 2025 Jan 7;16:440. doi: 10.1038/s41467-025-55841-x (PMC11704318; doi:10.1038/s41467-025-55841-x)
Supplement: Supplementary file 2 — Description of Additional Supplementary Files [file 41467_2025_55841_MOESM2_ESM.pdf]

Supplementary movie 1 | Dynamic transformation from the initial antivortex to vortex-antivortex pair driven by in-plane pulsed fields. $\mathbf{B}_0$ = 1250 Oe and w = 24 ps.

Supplementary movie 2 | Dynamic transformation from the initial antivortex to triple-domain driven by in-plane pulsed fields. $\mathbf{B}_0$ = 1250 Oe and w = 36 ps.
